# Association of public physical activity facilities and participation in community programs with leisure-time physical activity: does the association differ according to educational level and income?

**DOI:** 10.1186/s12889-022-12593-3

**Published:** 2022-02-11

**Authors:** André O. Werneck, Adewale L. Oyeyemi, Raphael H. O. Araújo, Luciana L. Barboza, Célia L. Szwarcwald, Danilo R. Silva

**Affiliations:** 1 Center for Epidemiological Research in Nutrition and Health, Department of Nutrition, School of Public Health, University of São Paulo (USP), Av. Dr. Arnaldo, 715 - Cerqueira César, São Paulo, SP 01246-904 Brazil; 2 Department of Physiotherapy, College of Medical Sciences, University of Maiduguri, Maiduguri, Borno State Nigeria; 3 Graduation Program in Health Sciences, Londrina State University, Londrina, Brazil; 4 Postgraduate Program in Physical Education, University of Brasília (UnB), Brasília, Brazil; 5 Instituto de Comunicação e Informação Científica e Tecnológica em Saúde (ICICT), Fundação Oswaldo Cruz (Fiocruz), Rio de Janeiro, Brazil; 6 Department of Physical Education, Federal University of Sergipe – UFS, São Cristóvão, Brazil

**Keywords:** Exercise, Sedentary lifestyle, Inequalities, Education, Built environment

## Abstract

**Background:**

Our aim was to analyze the association of the presence of public physical activity (PA) facilities and participation in public PA programs with leisure-time PA, with an emphasis on the moderating role of educational level and income.

**Methods:**

We used data of 88,531 adults (46,869 women), with a mean age of 47.2 ± 17.1y, from the 2019 Brazilian National Health Survey. Leisure-time PA (dichotomized considering 150 min/week), the presence of a public PA facility near the household (yes or no), participation in public PA programs (yes or no), educational level (divided into quintiles) and per capita income (divided into quintiles) were all self-reported through interviews. Adjusted logistic regression models were used for the analyses.

**Results:**

The presence of public PA facilities near the household and the participation in public PA programs were associated with higher leisure-time PA among all quintiles of income and educational level. However, multiplicative interactions revealed that participating in PA programs [Quintile (Q)1: OR: 13.99; 95%CI: 6.89–28.38 vs. Q5: OR: 3.48; 95%CI: 2.41–5.01] and the presence of public PA facilities near the household (Q1: OR: 3.07; 95%CI: 2.35–4.01 vs. Q5: OR: 1.38; 95%CI: 1.22–1.55) were more associated with higher odds of being active in the leisure-time among the lowest quintile of educational level.

**Conclusions:**

The presence of public PA facilities and participation in public PA programs are environmental correlates that may be relevant for designing effective public health interventions to reduce social inequalities in leisure-time PA among adults in low-income areas.

## Introduction

Physical activity (PA) is a protective factor for different health outcomes, including cardiovascular diseases, mental disorders, cancer and all-cause mortality [1, 2]. However, the proportion of adults who fail to reach the PA recommendation is elevated worldwide [3]. The health benefit of PA is domain specific [4]. Previous studies have shown that leisure-time PA presents the strongest associations with health outcomes, when compared with other domains such as transport and occupational PA [4–6].

Specifically in Brazil, leisure-time PA has increased over the years [7, 8], but this is mainly among people with higher educational level [8]. In this sense, the comprehension of factors that could reduce socioeconomic inequalities in leisure-time PA practice would help in the formulation of effective strategies for PA promotion across population sub-groups [9]. Considering the range of multi-level correlates of leisure-time PA, including individual (e.g., gender, age), interpersonal (e.g., social support), environmental (e.g., neighborhood walkability), regional and national policy (e.g., transport systems), and global (e.g., urbanization) [10], increasing opportunities for PA practice and participation is important for improving PA level, especially for the most vulnerable population groups [9]. Studies have found that several environmental correlates are associated with leisure-time PA, especially public PA facilities such as green areas and parks [10–12]. Also, community public programs to stimulate PA can increase leisure-time PA at the community level [13, 14]. In this sense, the presence of public areas for the PA practice and the participation in public PA programs reduces the barriers to the achievement of the PA guidelines, becoming a facilitator of opportunities, which can hypothetically be enhanced when considering people with lower income levels, who have less opportunities to practice PA.

However, the majority of the previous studies did not include nationally representative samples when testing the association of the presence of public PA facilities and participation in public PA programs with leisure-time PA. In addition, the role of income and educational level in this association is not clear. Therefore, we aimed to analyze the association of the presence of public PA facilities and participation in public PA programs with leisure-time PA, with an emphasis on the moderating role of educational level and income. We hypothesize that the presence of public PA facilities and participation in public PA programs will be associated with leisure-time PA and considering the lower opportunities among people with lower educational level and income, the presence of public PA facilities and participation in public PA programs could have a stronger association with leisure-time PA in these groups.

## Methods

### Sample

We used data from the Brazilian National Health Survey, which was a cross-sectional epidemiological study, conducted with a nationally representative sample of people ≥15 years old, during 2019 in Brazil. The sampling process was in three stages. Firstly, census tracts were randomly selected; next, households were randomly selected; and finally, in the households, one inhabitant (≥15 years old) was randomly selected. More details of the sampling process and weighting have been previously published elsewhere [15]. From the initial 100,541 selected households, 94,114 interviews were conducted. Due to missing data and excluding adolescents, the final sample was composed of 88,531 adults (≥18 years). Estimates were weighted considering the characteristics of the general population (i.e., sex and age group) as well as the non-response rate. The Brazilian Council of Ethics in Research approved all procedures according to the Helsinki declaration.

### Leisure-time PA

Leisure-time PA was self-reported based on a questionnaire previously validated for Brazilian adults [16]. The questionnaire is composed of specific questions asking about frequency and duration of leisure-time PA in habitual activities. For classification, total minutes of leisure-time PA was calculated and classified using the cutoff point of 150 min/week.

### Participation in public PA programs and public PA facilities near the household

The participation in public PA programs was assessed through the dichotomic questions (yes/no): “Do you know of any public program to encourage the practice of physical activity in your city?” and “Do you participate in this public program to encourage the practice of physical activity in your city?”. The presence of public PA facilities near the household was also assessed through the dichotomic question (yes/no): “Is there a public place close to your home (square, park, closed street, beach) to walk, exercise or play sports?”. The last two questions were adopted as exposures.

### Per capita income and educational level

The per capita income and educational level were categorized as quintiles. For the quintiles of income, we considered the household per capita income in monetary value and divided into quintiles, which the first quintile (Q1) represents the lowest level and the fifth quintile (Q5) the highest level. The classification values were (in Brazilian reais - BRL): Q1: ≤BRL417.00; Q2: ≥ BRL418.00 and ≤ BRL750.00; Q3: ≥ BRL750.00 and ≤ BRL1,099.00; Q4: ≥ BRL1,100.00 and ≤ BRL1,999.00; Q5: ≥ BRL2,000.00. For the quintiles of educational level, categories of highest educational achievement were grouped in order to have balanced groups in each survey (1- No education; 2- Primary incomplete; 3- Primary complete or incomplete high school; 4- High school; 5- More than high school), based on a previous study [17].

### Confounders

Gender, age group, TV-viewing, urban/rural and ethnicity were used as confounders in the analyzes. Age group was classified as 18-34y, 35-49y, 50-64y, and 65+. Ethnicity was assessed according to the self-reported skin color and classified as white, black, mixed, or other. TV-viewing was self-reported and classified using the cut-off point of ≥3 h/day.

### Statistical procedures

Characteristics of the sample were described using values of absolute and relative frequencies as well as 95% confidence interval. Logistic regression models were created to estimate the joint associations of participation in public PA programs and the presence of public PA facilities near the household with income and educational level in predicting leisure-time PA, adjusting for sex, age group, TV-viewing, urban/rural and ethnicity. In addition, multiplicative interactions were used to assess the moderation of income and educational level in the associations of participation in public PA programs and the presence of public PA facilities near the household with leisure-time PA. Also, the associations of participation in public PA programs and the presence of public PA facilities near the household with leisure-time PA stratifying by quintiles of per capita income and educational level were tested.

## Results

The final sample was composed of 88,531 adults (46,869 women), with a mean age of 47.2 ± 17.1 years. The characteristics of the sample according to leisure-time PA are presented in Table 1. The proportion of men, young adults, with higher educational level, higher income, PA facilities near home and participating in PA programs was higher among participants active in leisure-time.

**Table 1:** Characteristics of the sample according to leisure-time physical activity practice

		Whole sample		Active in leisure-time			
		*n* = 88,531		No (*n* = 66,278)		Yes (*n* = 22,253)	
		n	%	n	%	n	%
Gender	Male	41,662	46.8 (46.3–47.4)	30,985	45.7 (45.0–46.4)	10,677	50.1 (48.9–51.3)
	Female	46,869	53.2 (52.6–53.7)	35,293	54.3 (53.6–55.0)	11,576	49.9 (48.7–51.1)
Ethnicity	White	32,409	43.3 (42.7–43.9)	23,642	42.2 (41.6–42.9)	8767	46.1 (44.9–47.3)
	Black	10,132	11.5 (11.1–11.8)	7619	11.6 (11.2–12.0)	2513	11.1 (10.4–11.8)
	Mixed	44,646	43.8 (43.2–44.4)	34,023	44.7 (44.0–45.4)	10,623	41.3 (40.2–42.5)
	Other	1344	1.5 (1.3–1.6)	994	1.5 (1.3–1.7)	350	1.5 (1.2–1.8)
Type of residence	Urban	68,220	86.2 (85.9–86.5)	48,825	84.1 (83.7–84.5)	19,395	91.9 (91.4–92.3)
	Rural	20,311	13.8 (13.5–14.1)	17,453	15.9 (15.5–16.3)	2858	8.1 (7.7–8.6)
Age group	18–34	24,115	32.0 (31.4–32.5)	16,420	29.3 (28.6–29.9)	7695	39.4 (38.3–40.6)
	35–49	26,031	29.3 (28.8–29.8)	19,119	29.2 (28.5–29.8)	6912	29.7 (28.7–30.8)
	50–64	21,198	22.6 (22.1–23.1)	16,410	23.5 (22.9–24.0)	4788	20.3 (19.4–21.2)
	65+	17,187	16.1 (15.7–16.5)	14,329	18.1 (17.7–18.6)	2858	10.5 (9.9–11.2)
Educational level	Q1	7632	6.1 (5.9–6.3)	6910	7.4 (7.1–7.7)	722	2.6 (2.2–3.0)
	Q2	27,940	28.7 (28.2–29.2)	23,799	32.9 (32.3–33.5)	4141	16.9 (16.1–17.7)
	Q3	12,005	14.5 (14.1–14.9)	9339	15.2 (14.7–15.7)	2666	12.4 (11.6–13.2)
	Q4	23,378	29.8 (29.2–30.4)	16,409	28.3 (27.7–28.9)	6969	34.0 (32.8–35.2)
	Q5	17,576	21.0 (20.5–21.4)	9821	16.2 (15.7–16.7)	7755	34.2 (33.1–35.3)
Income	Q1	17,681	16.9 (16.5–17.3)	14,515	18.8 (18.3–19.3)	3188	11.7 (11.1–12.4)
	Q2	17,837	20.9 (20.5–21.4)	14,145	22.3 (21.7–22.8)	3692	17.2 (16.4–18.1)
	Q3	17,553	19.2 (18.8–19.7)	13,888	20.1 (19.5–20.6)	3665	16.9 (16.0–17.8)
	Q4	17,716	22.7 (22.2–23.2)	13,027	22.3 (21.7–22.9)	4667	23.7 (22.6–24.8)
	Q5	17,744	20.3 (19.8–20.7)	10,710	16.6 (16.1–17.1)	7034	30.5 (29.4–31.6)
TV-viewing	< 3 h	69,282	78.2 (77.7–78.7)	51,278	77.4 (76.9–78.0)	18,004	80.5 (79.4–81.5)
	≥3 h	19,249	21.8 (21.3–22.3)	15,000	22.6 (22.0–23.1)	4249	19.5 (18.5–20.6)
PA programs participation	No	86,169	97.3 (97.1–97.5)	65,287	98.4 (98.3–98.6)	20,882	94.0 (93.5–94.5)
	Yes	2362	2.7 (2.5–2.9)	991	1.6 (1.4–1.7)	1371	6.0 (5.5–6.5)
PA facilities near household	No	45,201	56.2 (55.6–56.8)	30,670	47.7 (47.0–48.4)	14,531	67.1 (66.0–68.3)
	Yes	43,330	43.8 (43.2–44.4)	35,608	52.3 (51.6–53.0)	7722	32.9 (31.7-34.0)

*Note*. Values of relative frequencies are weighted. Q, quintile. Both quintiles of income and educational level are based on the distribution of the sample into quintiles. As it was not possible to estimate the number of educational years, the closest categorization of quintiles was: Q1- No education; Q2- Primary incomplete; Q3- Primary complete or incomplete high school; Q4- High school; Q5- More than high school

**Fig. 1:**
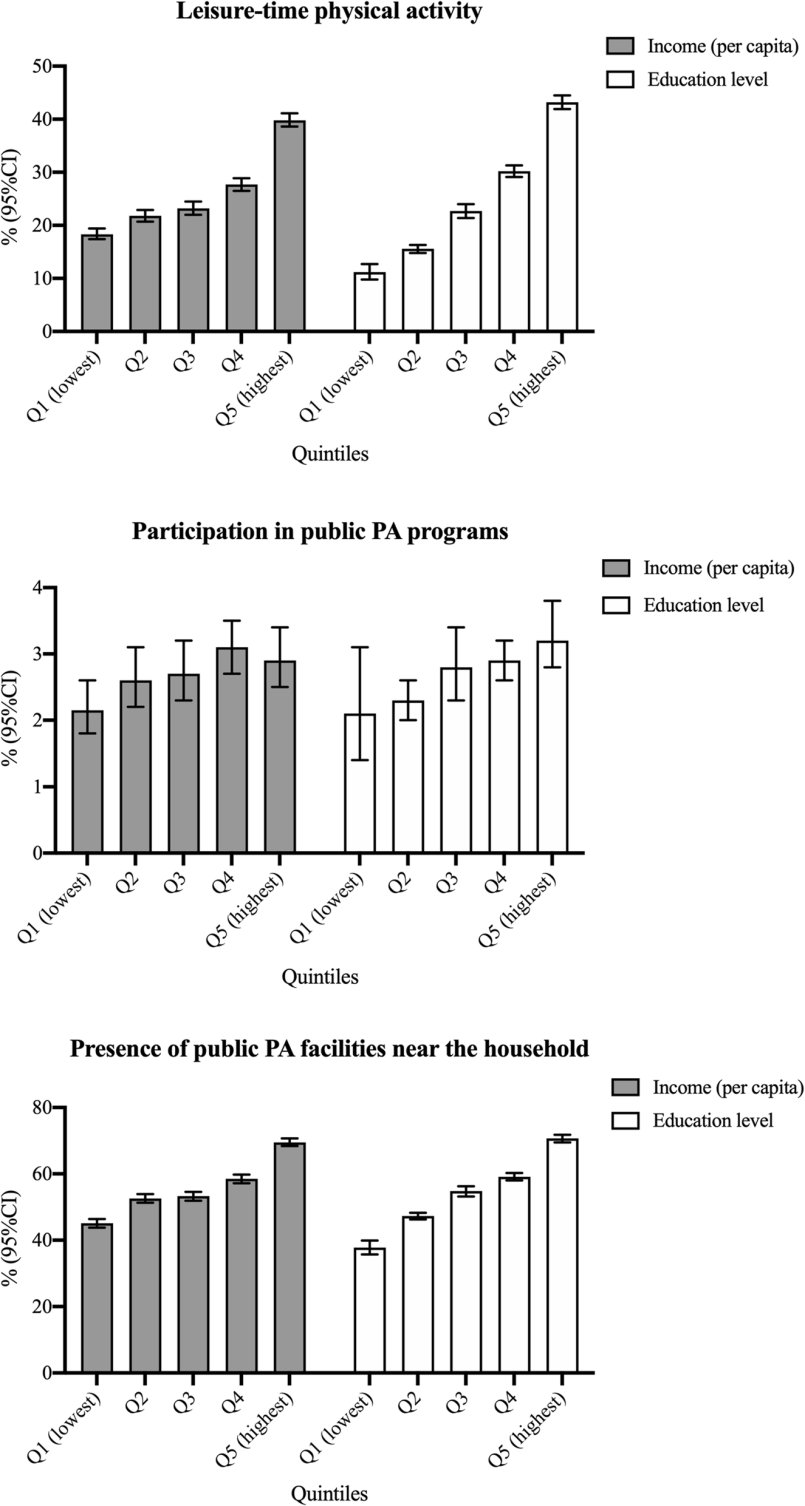
The prevalence of leisure-time PA, participation in public PA programs and the presence of public PA facilities near the household according to quintiles of educational level and income. Note. PA, physical activity. Q, quintile. Both quintiles of income and educational level are based on the distribution of the sample into quintiles. As it was not possible to estimate the number of education years, the closest categorization of quintiles was: Q1- No education; Q2- Primary incomplete; Q3- Primary complete or incomplete high school; Q4- High school; Q5- More than high school

Figure 1 shows the prevalence of leisure-time PA, participation in PA programs and presence of public PA facilities near the household according to quintiles of per capita income and educational level. Higher quintiles of income and educational level presented a higher prevalence of leisure-time PA and presence of public PA facilities near the household. The participation in PA programs was similar according to the quintiles of income and educational level.

**Fig. 2:**
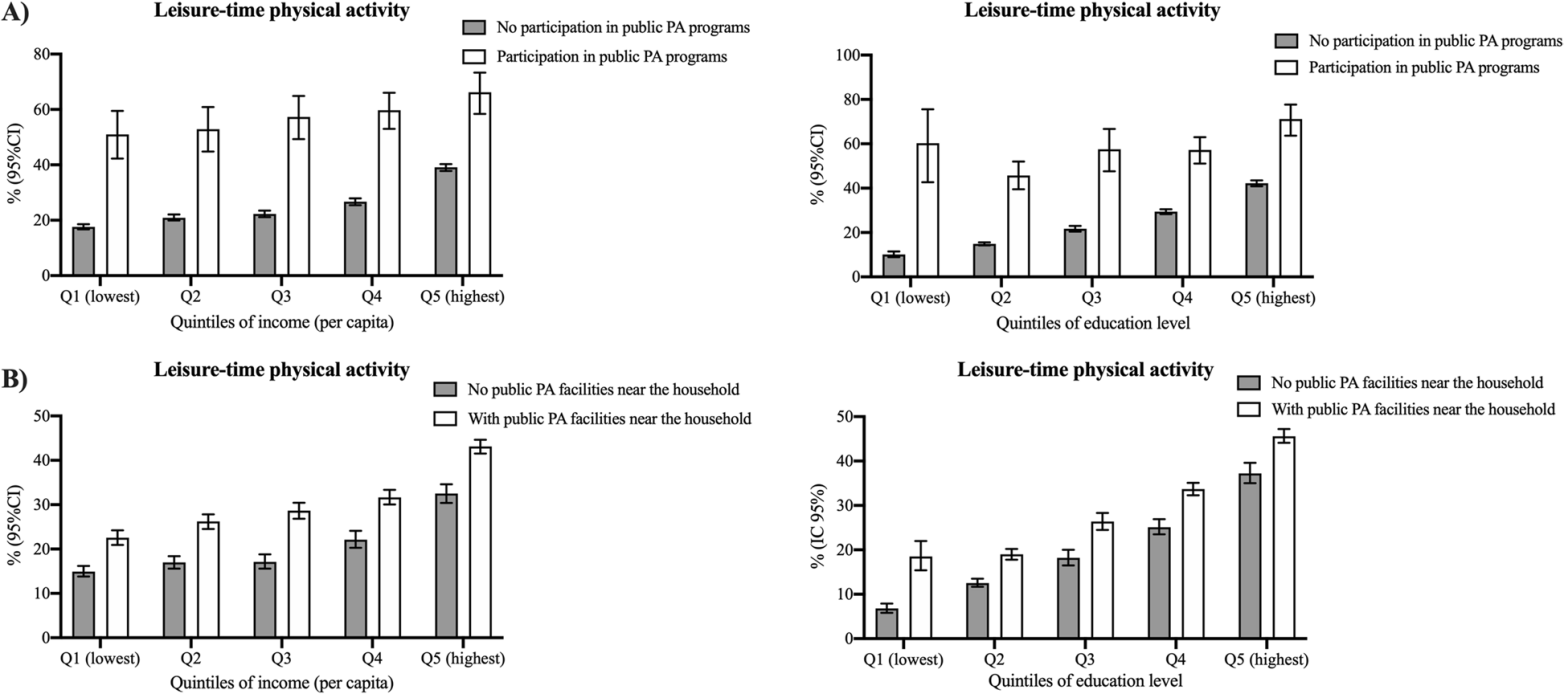
Prevalence of ≥150 min/week of leisure-time physical activity according to quintiles of educational level and income as well as the prevalence of physical activity public programs and public PA facilities. Note. PA, physical activity. Q, quintile. Both quintiles of income and educational level are based on the distribution of the sample into quintiles. As it was not possible to estimate the number of education years, the closest categorization of quintiles was: Q1- No education; Q2- Primary incomplete; Q3- Primary complete or incomplete high school; Q4- High school; Q5- More than high school

Figure 2 shows the prevalence of leisure-time PA according to quintiles of income and educational level as well as participation in PA programs and the presence of facilities near the household. The prevalence of leisure-time PA was higher among participants who engaged in PA programs across all the quintiles of income and educational level. Similarly, the prevalence of leisure-time PA was higher among participants with public PA facilities near the household. However, this was more pronounced for participants in the lowest quintile of educational level, in which the prevalence of leisure-time PA was more than 100% higher among those with facilities near the household (18.5% vs. 6.8%).

The combined associations of educational level and income with PA programs and public PA facilities near the household in the association with leisure-time PA are presented in Table 2. Multiplicative interactions revealed that participating in PA programs (Quintile 1: OR: 13.99; 95%CI: 6.89–28.38 vs. Quintile 5: OR: 3.48; 95%CI: 2.41–5.01) and the presence of public PA facilities near the household (Quintile 1: OR: 3.07; 95%CI: 2.35–4.01 vs. Quintile 5: OR: 1.38; 95%CI: 1.22–1.55) were more associated with lower odds of engaging in leisure-time PA in the lowest quintile of educational level.

**Table 2:** Joint associations of educational level and income with physical activity programs and public physical activity facilities near the household in the association with leisure-time physical activity

	Joint OR (95%CI)	Multiplicative interaction OR (95%CI)	Stratified OR (95%CI)
**Educational level + PA programs**			
Q5 + No	REF	REF	REF
Q5 + Yes	3.48 (2.42–5.00)	–	3.48 (2.41–5.01)
Q4 + No	0.57 (0.52–0.61)	–	REF
Q4 + Yes	1.96 (1.52–2.51)	0.99 (0.64–1.54)	3.52 (2.74–4.51)
Q3 + No	0.38 (0.35–0.42)	–	REF
Q3 + Yes	1.98 (1.33–2.93)	1.48 (0.86–2.53)	5.35 (3.59–7.96)
Q2 + No	0.28 (0.26–0.30)	–	REF
Q2 + Yes	1.42 (1.09–1.85)	1.46 (0.94–2.29)	4.80 (3.69–6.24)
Q1 + No	0.20 (0.17–0.23)	–	REF
Q1 + Yes	2.81 (1.35–5.84)	4.06 (1.78–9.26)	13.99 (6.89–28.38)
**Income + PA programs**			
Q5 + No	REF	REF	REF
Q5 + Yes	3.34 (2.33–4.78)	–	3.26 (2.27–4.68)
Q4 + No	0.55 (0.50–0.60)	–	REF
Q4 + Yes	2.53 (1.86–3.42)	1.38 (0.86–2.22)	4.50 (3.31–6.10)
Q3 + No	0.45 (0.41–0.49)	–	REF
Q3 + Yes	2.43 (1.70–3.45)	1.62 (0.98–2.68)	5.67 (3.94–8.17)
Q2 + No	0.38 (0.35–0.41)	–	REF
Q2 + Yes	1.72 (1.22–2.42)	1.36 (0.83–2.23)	4.55 (2.23–6.39)
Q1 + No	0.30 (0.27–0.33)	–	REF
Q1 + Yes	1.43 (1.01–2.00)	1.42 (0.86–2.33)	5.16 (3.69–7.22)
**Educational level + facilities**			
Q5 + No	REF	REF	REF
Q5 + Yes	1.39 (1.24–1.56)	–	1.38 (1.22–1.55)
Q4 + No	0.56 (0.49–0.64)	–	REF
Q4 + Yes	0.83 (0.74–0.93)	1.06 (0.90–1.24)	1.46 (1.31–1.64)
Q3 + No	0.38 (0.32–0.44)	–	REF
Q3 + Yes	0.59 (0.51–0.68)	1.12 (0.92–1.36)	1.56 (1.32–1.83)
Q2 + No	0.28 (0.24–0.32)	–	REF
Q2 + Yes	0.43 (0.38–0.49)	1.12 (0.95–1.32)	1.59 (1.40–1.79)
Q1 + No	0.15 (0.13–0.19)	–	REF
Q1 + Yes	0.45 (0.35–0.57)	2.10 (1.56–2.83)	3.07 (2.35–4.01)
**Income + facilities**			
Q5 + No	REF	REF	REF
Q5 + Yes	1.49 (1.32–1.67)	–	1.44 (1.28–1.62)
Q4 + No	0.57 (0.49–0.66)	–	REF
Q4 + Yes	0.86 (0.76–0.98)	1.02 (0.85–1.22)	1.49 (1.30–1.71)
Q3 + No	0.44 (0.38–0.51)	–	REF
Q3 + Yes	0.76 (0.66–0.86)	1.16 (0.89–1.27)	1.73 (1.48–2.01)
Q2 + No	0.39 (0.34–0.45)	–	REF
Q2 + Yes	0.61 (0.54–0.70)	1.06 (0.89–1.27)	1.60 (1.40–1.84)
Q1 + No	0.32 (0.28–0.37)	–	REF
Q1 + Yes	0.49 (0.42–0.56)	1.02 (0.85–1.22)	1.59 (1.38–1.84)

*Note*. Adjusted for sex, age group, TV-viewing, urban/rural and ethnicity. *OR* odds ratio, *CI* confidence interval. Q, quintile. Both quintiles of income and educational level are based on the distribution of the sample into quintiles. As it was not possible to estimate the number of education years, the closest categorization of quintiles was: Q1- No education; Q2- Primary incomplete; Q3- Primary complete or incomplete high school; Q4- High school; Q5- More than high school

## Discussion

We aimed to investigate the association of the presence of public PA facilities near the household and participation in public programs of PA with leisure-time PA as well as whether income and educational level could moderate this association, using a nationally representative sample of Brazilian adults. Our main findings were that the presence of public PA facilities near the household and participation in public PA programs were associated with higher leisure-time PA among all quintiles of income and educational level. However, these associations were more pronounced among the lowest quintiles of educational level and income.

Our findings confirm that the presence of public PA facilities near the household and participation in public PA programs are associated with a higher leisure-time PA practice as reported in previous findings [12, 13, 18, 19]. Although both correlates were associated with higher leisure-time PA across all quintiles of income and educational level, the association was stronger in the lowest educational level group. These findings highlight that PA policies should be more decisive for PA promotion among the poorest as the opportunities for PA practice are lower in this group [9].

There are marked PA inequalities especially in the leisure-time domain, in which people with higher educational level and income present higher PA practice [7, 8]. In this sense, the increases in leisure-time PA levels over the years in Brazil is somewhat contrasting with the also increasing socioeconomic inequalities in the practice of leisure-time PA [8]. Considering the context of inequalities in leisure-time PA and the more decisive role of public PA facilities and participation in public PA programs, it is noteworthy that a higher proportion of people in the highest quintile of educational level and income reported the presence of public PA facilities near the household. This finding is consistent with previous Brazilian finding [20] as well as from other countries [21] and underscore the urgent need to address disparity and inequality in presence of PA facilities in low-income areas and disadvantaged regions of Brazil.

Our findings highlight that the building and revitalization of public PA facilities such as parks and recreational centers for PA practice need to be prioritized in areas with lower socioeconomic development. Despite the association with PA, the proximity to public PA facilities could also be positive for well-being and quality of life [22]. However, a frequent consequence of revitalization is the increasing gentrification and hygienisation in the surrounding areas, which could cause an urban displacement of people with lower socioeconomic conditions from the revitalized areas to areas without public facilities [23, 24]. Some actions could help to avoid gentrification, such as involving the community in the planning of the revitalizations as well as creating measures to avoid the real estate speculation throughout the surrounding areas [25].

Also, the expansion of community health programs for the stimulation of PA should be prioritized, especially in deprived areas. Although Brazil has a large program that includes PA professionals in primary health care (Multidisciplinary Primary Care Teams), the distribution of the units with PA interventions is unequal considering different geographical regions [26]. The supervised PA promoted by the different programs already proved to be effective, even in highly deprived areas, such as in “*favelas*” [14, 27]. Also, both the building of public PA facilities and the stimulation of community health programs for the stimulation of PA should be taken together. For example, analyzing data from the same Brazilian National Survey (adjusting for the same confounders), participants living in areas with a public PA facility near the household were 268% (OR:3.69; 95%CI:3.03–4.50) more likely to participate in public PA programs, highlighting that most of the programs occurs in public PA facilities. In this sense, building PA facilities in more deprived regions would also contribute to the expansion and engagement of people with lower socioeconomic status in public PA programs. In addition, considering the difference observed between the frequency of public PA facilities, leisure-time PA and participation in public PA programs across categories of educational level and income, further investigations are needed in order to identify strategies to optimize the use of public PA facilities.

The strength of our study is including a large nationally representative sample of Brazilian adults with data on PA, proximity to public PA facilities, income and educational level. However, our findings should be inferred in light of possible limitations. First, our study has a cross-sectional design and causal relations should be avoided. Second, our findings were based on self-reported measures, which can present bias. Third, the levels of participation in public PA programs were considerably low, which is a reflection of the low coverage of health programs involving physical education professionals, which may have reduced the sampling power. However, up to the moment, there is no feasible method to estimate domains of PA in large population studies. Also, both perceived and objectively-assessed built environmental characteristics are associated with PA [11].

In conclusion, we found participation in public PA programs and presence of public PA facilities near the household to be associated with higher leisure-time PA and the associations are stronger in the group with lower educational level. Our findings suggest that presence of public PA facilities and participation in public PA programs are built environmental correlates that could be relevant for designing effective public health intervention for reducing social inequalities in leisure-time PA among adults in low-income areas of Brazil. Future studies should evaluate whether revitalization and the building of open spaces in areas of lower socioeconomic conditions could increase leisure-time PA among people with lower socioeconomic status.

## Data Availability

Data from the National Health Survey is available in the Brazilian Institute of Geography and Statistics website (https://www.ibge.gov.br/en/home-eng.html).
